# Refining Time-Activity Classification of Human Subjects Using the Global Positioning System

**DOI:** 10.1371/journal.pone.0148875

**Published:** 2016-02-26

**Authors:** Maogui Hu, Wei Li, Lianfa Li, Douglas Houston, Jun Wu

**Affiliations:** 1 Program in Public Health, College of Health Sciences, University of California Irvine, Irvine, California, United States of America; 2 Department of Landscape Architecture and Urban Planning, Texas A&M University, College Station, Texas, United States of America; 3 Department of Planning, Policy and Design, School of Social Ecology, University of California Irvine, Irvine, California, United States of America; 4 State Key Laboratory of Resources and Environmental Information System, Institute of Geographic Sciences and Natural Resources Research, CAS, Beijing, China; 5 Jiangsu Center for Collaborative Innovation in Geographical Information Resource Development and Application, Nanjing, China; University of Bremen, GERMANY

## Abstract

**Background:**

Detailed spatial location information is important in accurately estimating personal exposure to air pollution. Global Position System (GPS) has been widely used in tracking personal paths and activities. Previous researchers have developed time-activity classification models based on GPS data, most of them were developed for specific regions. An adaptive model for time-location classification can be widely applied to air pollution studies that use GPS to track individual level time-activity patterns.

**Methods:**

Time-activity data were collected for seven days using GPS loggers and accelerometers from thirteen adult participants from Southern California under free living conditions. We developed an automated model based on random forests to classify major time-activity patterns (i.e. indoor, outdoor-static, outdoor-walking, and in-vehicle travel). Sensitivity analysis was conducted to examine the contribution of the accelerometer data and the supplemental spatial data (i.e. roadway and tax parcel data) to the accuracy of time-activity classification. Our model was evaluated using both leave-one-fold-out and leave-one-subject-out methods.

**Results:**

Maximum speeds in averaging time intervals of 7 and 5 minutes, and distance to primary highways with limited access were found to be the three most important variables in the classification model. Leave-one-fold-out cross-validation showed an overall accuracy of 99.71%. Sensitivities varied from 84.62% (outdoor walking) to 99.90% (indoor). Specificities varied from 96.33% (indoor) to 99.98% (outdoor static). The exclusion of accelerometer and ambient light sensor variables caused a slight loss in sensitivity for outdoor walking, but little loss in overall accuracy. However, leave-one-subject-out cross-validation showed considerable loss in sensitivity for outdoor static and outdoor walking conditions.

**Conclusions:**

The random forests classification model can achieve high accuracy for the four major time-activity categories. The model also performed well with just GPS, road and tax parcel data. However, caution is warranted when generalizing the model developed from a small number of subjects to other populations.

## Background

Environmental air pollution has been associated with a variety of adverse health outcomes, including respiratory illness, cardiovascular diseases, pregnancy outcomes, and morbidity [1–5]. The knowledge of where individuals spend time is essential for human exposure assessment of air pollution because air pollutant concentrations may vary significantly by location. Studies have shown that traffic-generated air pollutants such as ultrafine particles can be up to ten times higher inside a vehicle compared to ambient outdoor concentrations because of proximity to vehicle exhaust [6–8]. Outdoor walking and cycling are often associated with lower concentrations of traffic-related pollutants than in-vehicle commuting [9], but likely correspond with increased inhalation rates and longer travel durations, which can result in a higher dose of air pollutant inhalation [10, 11]. In addition, air pollutant concentrations can be much higher indoors than outdoors for pollutants with predominant indoor sources (e.g. environmental tobacco smoke) and vice versa for pollutants with predominant outdoor sources (e.g. ozone) [12, 13]. Our previous personal exposure measurement study reported that in-vehicles travel time explained approximately 40% of the variance in daily personal exposure to particle-bound polycyclic aromatic hydrocarbon [14]. Accurate characterization of people’s time-activity patterns can significantly reduce errors in exposure estimates in environmental epidemiological studies in which personal exposure is not measured directly and has to be estimated.

Global Positioning System (GPS) techniques have been increasingly used to track people’s time-activity or commuting patterns [15–20]. Compared to the conventional methods for time-activity collections (e.g. self-reported paper diary and telephone interview), GPS tracking has the advantages of continuous recording, high temporal resolution, and minimum reporting burden for participants [21]. However, barriers exist for accurately extracting time-activity patterns for human subjects from raw GPS data because they are not consistently reliable due to errors caused by satellite or receiver issues, atmospheric and ionospheric disturbances, multipath signal reflection, or signal loss or blocking [22]. The multipath problem occurs mainly in urban areas where tall buildings and structures reflect satellite signals many times before they reach a GPS device, leading to GPS coordinate errors [23]. In fact, few air pollution epidemiological studies have effectively used GPS data to classify time-activity patterns, likely due to issues including the quality of GPS data, the compliance of human subjects, and the lack of reliable methods to mine raw GPS data [24]. GPS data classification techniques have been largely documented in the travel behavior and physical activity research fields. Travel modes, activity places, and trip-end detection were the main application of GPS classification algorithms [25, 26]. A number of studies have developed methods to classify travel activity using GPS data or the combination of GPS and body-worn sensors [27–35]. Ellis et al. (2013, 2014) adopted body-worn sensors, e.g. accelerometer and SenseCam camera, to classify the physical activities into five modes (bicycling, riding in a vehicle, sitting, standing, and walking/running) with a random forest classification model [36, 37]. Based on multinomial logistic regression model, Kohla et al. (2014) used both GPS and acceleration data to predict eight modes of transport [38]. Combining GPS, geographical information system, and accelerometer data, Brondeel et al. (2015) developed a trip-level transportation mode prediction model based on the random forest method, and obtained 90% correct prediction [39]. However, the reliability of accelerometer data can be negatively affected by external factors, such as clothing and weather change [40, 41]. Spatial data (e.g. roads and building rooftop) have also been used to improve the accuracy of time-activity classification. Wu et al. (2011) used roadway data to build decision rules to classify GPS points [42]. Nethery et al. (2014) manually plotted the boundary of participants’ home and school buildings to help detect specific locations [43]. The method was also used by Breen et al. (2014) and proved to be efficient in estimating time-activities of individuals [44].

This paper aims to develop an adaptive time-activity classification methodology with raw GPS data and publicly-available spatial data, and to evaluate the effectiveness of incorporating accelerometer data in the model. With this methodology, researchers can generate time-activity patterns based on data collected from different geographical regions and study settings. We focused on four major time-activity categories (i.e. indoor, outdoor static, outdoor walking, and in-vehicle travel), which are important in determining air pollutant exposure in urban populations.

## Method

### Study Participants

A convenience sample of thirteen students and staff from the University of California, Irvine participated in this study from May to July, 2012. The subjects were informed that the participation was voluntary; they were aware that their GPS traces and physical activity levels would be recorded and analyzed, and their confidentiality would be strictly protected. They submitted a written informed consent form before data collection started. The study protocol and materials were approved by the University of California, Irvine Institutional Review Board for social/behavioral research.

### Data Collection

Time-stamped location data were recorded with two GPS devices, BT-Q1000XT (QSTARZ^TM^; approximately 65 g) and VGPS-900 (Visiontac^TM^; approximately 55 g). These two GPS devices were used because of their complementary features. VGPS-900 was capable of voice tagging points of interest and activity changes, but it had a relatively short battery life of about 18 hours [45]. BT-Q1000XT had a longer battery life up to 48 hours, but had no voice recording capability [45]. Physical activity level was monitored using an Actigraph GT3X+ accelerometer (ActiLife^TM^; 19 grams), in which a light sensor was built. The light sensor detects ambient light in lumens per square meter (lux), a measure of light intensity. Indoor lighting typically ranges from 50 to 500 lux and outdoor light ranges from 1000 lux on an overcast day to 130,000 lux in direct sunlight [46]. The study participants were instructed to wear the two GPS units and the accelerometer continuously for 5 weekdays and 2 weekend days. They were asked to take the devices off right before going to bed and put them on first thing every morning. The GPS devices needed to be charged every night, while no battery charge was required for the accelerometer since it had a battery life of approximately 20 days. We asked the participants to attach the accelerometer to the belt around the waist just above the right hipbone and carry the GPS devices in his/her pocket or bag/purse. Instead of taking time-activity diaries, we asked the subjects to record any changes in activity, mode of transportation, and/or activity levels in real time using the voice recording function on the VGPS-900 device. For example, a recorded sentence could be “exiting building, start walking, and moderate activity” or “stop walking, entering vehicle, start driving, sedentary.” The GPS devices were initialized using the QTravel software and set to record data at 15-second intervals, while the accelerometers were initialized using ActiLife at 30-second epochs and after the computer clock was synchronized to coordinated universal time used by the GPS.

Besides the GPS and accelerometer data, we also collected land-use and roadway data for the study area. The typical surroundings of these participants were multifamily housing complexes, office buildings, suburban shopping plazas and the roadways connecting the above locations. Parcel-level land-use data in 2008 were obtained from the Southern California Association of Government. There were four major land-use categories with 25 sub-categories, from which we extracted residential and commercial/services sub-categories from the urban or built-up category. The roadway data were obtained from the ESRI StreetMap^TM^ North America bundled with ArcGIS 10.1 software.

### Data quality checking and preprocessing

Each subject submitted a diary transcribed from time-stamped VGPS-900 voice logs in Excel spreadsheet when returning the GPS and accelerometer devices to the research staff. Problems encountered during the sampling period were also recorded in the spreadsheet. Typical problems included (1) GPS device ran out of battery; (2) GPS device was left inside car; and (3) omitted or delayed voice recording when activity and/or location changed. Subjects were instructed to recall and correct the time of location/activity changes in the spreadsheet when these problems occurred.

The GPS data points and voice records were linked for each subject according to the GPS data time stamp in SAS 9.2 (SAS Institute Inc., Cary, NC). All GPS points with voice-logged activity were then displayed in ArcGIS (ESRI, Redlands, CA) for additional data quality checks. Consistent with the previous studies, we identified and removed 307 erroneous GPS points with abrupt position change that resulted in a speed of more than 200 km/h in 15 seconds [42, 43]. We then identified GPS points (e.g. about 3%) that were implausibly labeled, which were likely caused by missing voice logs. For these points, we analyzed their speed and distances to roadways and parcels with land-use information, and the time-activity patterns immediately before and after these points in time. For example, GPS points within a parcel with low speed (less than 3 km/h) were assumed to be indoors. Points on roads with a speed faster than 15 km/h were assumed to be in-vehicle travel. Points that were outside a parcel, did not move or moved slowly (speed less than 0.5 km/h), and lasted longer than 2 minutes were treated as outdoor static. Otherwise, the points were assumed to be outdoor walking. Two out of the thirteen participants biked and two traveled by bus and light rail, respectively. We excluded those non-vehicle travel points from the four participants because of the limited sample size for these travel modes. Finally, based on the voice records and the interactive visualization and analysis in ArcGIS, each GPS point was manually labeled as one of the four major time-activity categories: indoor (including home and other indoor locations), outdoor static, outdoor walking, and in-vehicle travel.

### Random forests model

**Fig 1:**
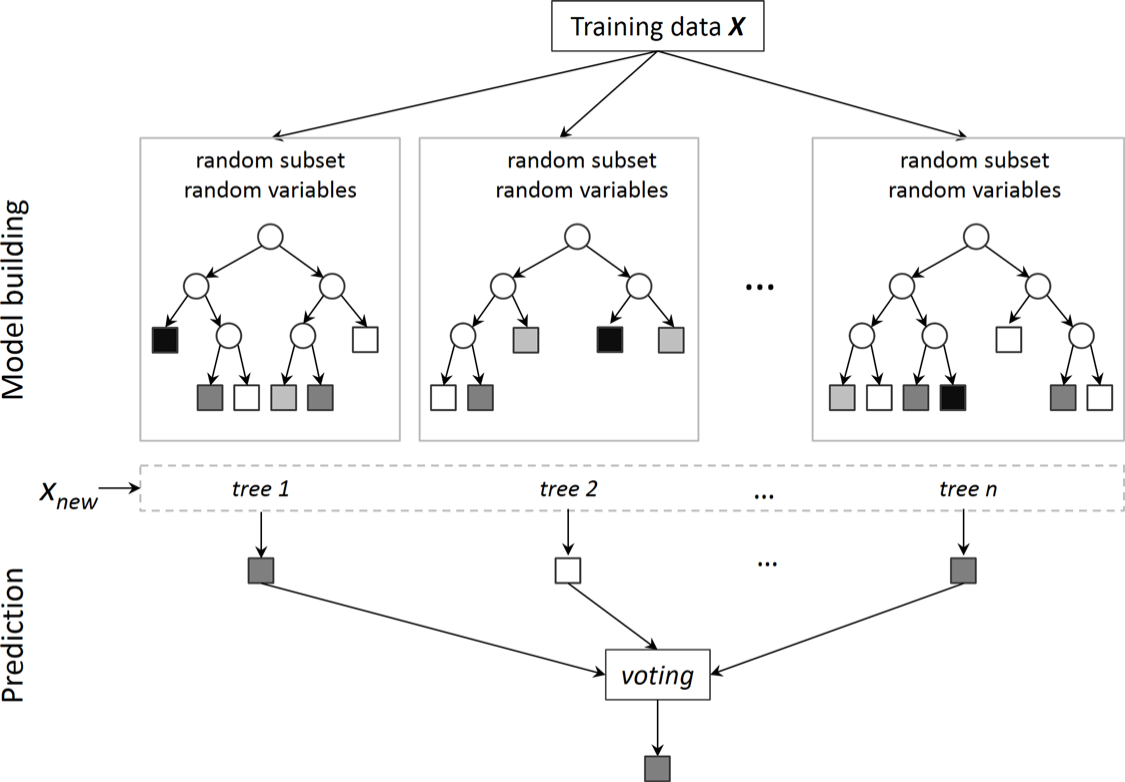
Random forests classification.

The random forests model is a classification and regression method based on decision trees [47]. It is one of the most efficient algorithms in machine learning, and has been widely used in many domains, including environment [48, 49], ecology [50], bioinformatics [51, 52] and remote sensing [53, 54]. The random forests model has also been used in physical time-activity classification in previous studies. Ellis et al. (2013) predicted five physical activity classes (bicycling, riding, sitting, standing, and walking/running) based on the random forests model [36]. Ellis et al. (2014) developed a random forests model to predict four physical activities classes (household, stairs, walking, and running) with hip accelerometer, and achieved an average accuracy of 92.3% [37]. Random forests is different from the traditional decision tree classification in two main aspects, i.e. (1) it consists of an ensemble of decision trees; (2) each tree is trained separately by a collection of randomly selected samples from the data [47]. Generally, random forests classification contains two main steps (Fig 1): (1) training step: for a given number of trees *N*, *N* bootstrap samples are generated independently from the input data; for each bootstrap sample set a decision tree is trained by a subset of randomly selected predictor variables and fully grown with no pruning; (2) predicting step: prediction of new data other than the input data with the trained trees. Each record in the new data gets a class from each tree, while the most voted class is selected as the final class of the record. In the training step, a typical bootstrap sample contains about 63% (1—e^-1^ ≈ 63%) of the input dataset; the remaining 37% of the data, called out-of-bag, are used to calculate the error rate [55, 56]. In addition to high prediction accuracy, random forests model is robust to outliers and noise, runs efficiently on large data, and can capture non-linear associations without specifying the underlying model. However, it is not straightforward to interpret the decision rules, which might be one of the biggest shortcomings. Temporal autocorrelation between points was not considered in the model. Furthermore, the decision tree models are primarily data driven, thus the quality of training data is critical for the development of reliable and stable models. Although we strive to procure the data with the highest quality, misclassifications were inherited in the data because of the imperfect data collection method. In this study, random forests was used to classify major time- activity patterns. We ran the model using the package *randomForest* in *R* [55, 57].

### Predictor variables

#### Roadway-related variables

Four categories of roads were extracted: primary highway with limited access (Census Feature Class Codes (FCC) = A1), primary highways without limited access (FCC = A2), secondary and connecting road (FCC = A3), and local, neighbor, and rural road (FCC = A4).

#### Distance from road and parcel data

GPS points from BT-Q1000XT were used to calculate the distance, speed and direction variables. For each GPS point, Euclidean distances were calculated to the nearest residential and commercial/services parcels, as well as highways and local roads.

#### Speed and direction from GPS data

To account for potential errors in GPS data caused by poor satellite signal reception (particularly in or near buildings) [22, 23], two types of speed variables were calculated by four averaging time intervals (i.e. 1, 3, 5 and 7 minutes). Speed based on accumulated distance (type-I speed) was estimated for each GPS point by consecutively summing the distance of every 15 seconds GPS point pairs within a specific averaging time window prior to this point, and dividing the total distance by the average time. A second measure of speed (type-II speed) was also calculated for the GPS point by dividing the straight line distance between the first and the last point by the specific averaging time. Besides the two types of speed, maximum speed, minimum speed, and the variance of speed during the specific time intervals were also calculated.

**Fig 2:**
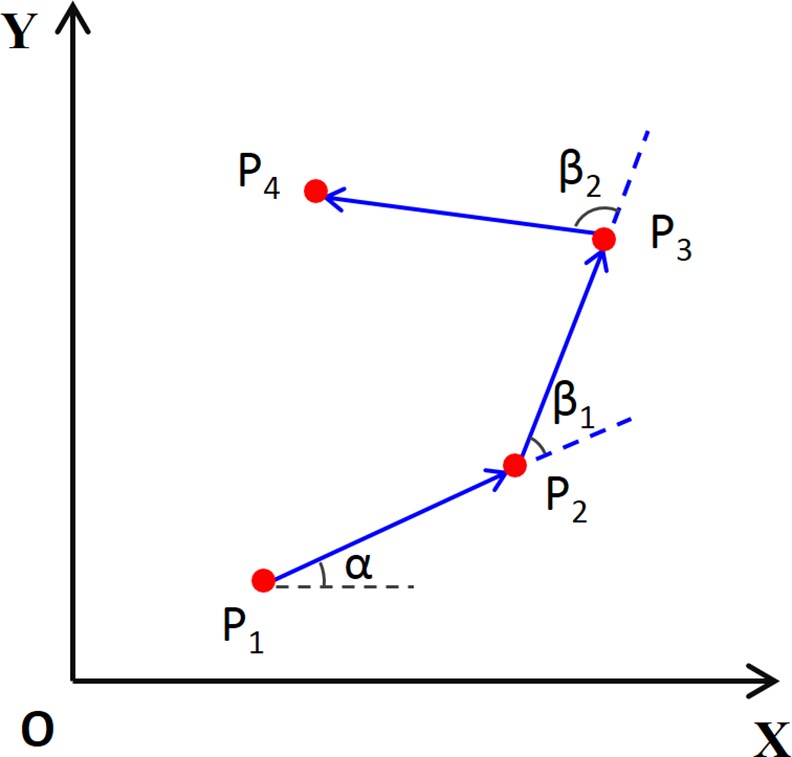
Direction and direction change of GPS data pairs.

*Direction* of GPS points: For each 15 seconds GPS data pair, we calculated a direction vector with the counter-clock angle relative to the east. The difference of directions was the direction change between two contiguous 15 seconds GPS data pairs (two pairs were shown in Fig 2: P_1_P_2_—P_2_P_3_, and P_2_P_3_—P_3_P_4_). Similar to speed, two types of direction changes were calculated for the four averaging times. An average direction change was estimated by consecutively summing direction changes within a specific averaging time prior to the specific GPS point. Direction can be strongly influenced by the drifting of GPS points, which is more serious under relatively poor satellite signals (e.g. indoor environment). We calculated a second measure of direction by assuming that if the distance between two contiguous GPS points was less than 2.5 m (the spatial accuracy specified by the GPS device manufacture), the two points were identical thus the direction change was zero. Sensitivity analysis showed that the second direction measure was better than the first direction measure in decreasing the classification error of GPS points.

#### Physical activity from accelerometer data

The accelerometer data included six key variables: acceleration in three axes (vertical, horizontal and perpendicular respectively), step counts, ambient light intensity and orientation of the device. The step counts were calculated based on the data collected on the vertical axis. The inclination of the device included subject standing, subject lying horizontal and subject sitting.

#### Variable selection

Including a limited number of key variables not only decreases the burden of variable preparation but also reduces the calculation time, especially for large memory-required algorithms [58, 59]. Variable importance is a special characteristic of random forests that distinguishes it from the other decision tree models. Variable importance can be measured by the mean decrease in accuracy (MDA), a permutation-based accuracy measurement method. After the random forests model was trained with all variables, two out-of-bag errors were calculated and compared to measure the importance of a variable. The first out-of-bag error was obtained by comparing the predicted classification of the out-of-bag samples with the random forests model and the real classification of the out-of-bag samples. Afterwards, data of the selected variable of the out-of-bag samples were permuted. Then, a second out-of-bag error was calculated with the permuted out-of-bag data and the same random forests model. Next, the difference of the two errors was calculated as the MDA of the variable and averaged from all trees. In this study, the process was repeated 200 times for each variable to obtain stable results. Finally, the relative importance of all the variables was obtained by ranking the MDAs.

### Model parameter tuning

The random forests model requires two major parameter inputs, namely the number of trees and the number of candidate variables randomly selected at each split. Although a higher number of trees will likely improve model performance, it is computationally intensive for the large number of GPS points. We conducted sensitive analysis and identified the optimal number of trees when the classification error became small and stable.

The default value of the number of candidate predictors is the square root of the number of input predictors. We increased the number of candidate predictors gradually to find the lowest number when the classification error was small and stable.

### Sensitivity analysis

Sensitivity analysis was conducted by excluding accelerometer variables to assess whether the incorporation of accelerometer variables significantly improved the model performance. Similarly, we examined the effectiveness of supplemental spatial variables (i.e. distance to road and residential and commercial/services parcels).

### Model validation

Model evaluation was conducted based on two validation methods, leave-one-fold-out and leave-one-subject-out [60]. The leave-one-fold out method randomly splits input data into 10 equal-sized subsets. Each time, 9 subsets were used to train the model and the remaining subset was used for model evaluation. The process was iterated 10 times until each sample has been used for model evaluation. For each iteration of the leave-one-subject-out approach, we excluded data from one participant completely for model evaluation, and use data from the remaining participants to train the model. The process was iterated 13 times until data for every participant have been used for model evaluation. For each method, the results from all iterations were grouped together to calculate the mean validation results. Model performance was quantified by three measures. The first is sensitivity, indicating the ability of the model to identify specific cases. It is calculated as the number of “true positive” divided by the sum of the number of “true positive” and the number of “false negative”, where true positive means the specific cases are correctly predicted by the model; false negative means the specific cases are incorrectly predicted as non-cases). The second is specificity, indicating the ability of the model to identify non-cases. It is calculated as the number of “true negative” divided by the number “true negative” and the number of “false positive”, where the true negative means non-cases are correctly predicted as non-cases; false positive means non-cases are incorrectly predicted as the special cases). The third is accuracy, indicating the proportion of predicted cases correctly classified. It is calculated as the sum of the number of “true positive” and the number of “true negative” divided by the number of all estimation. The out-of-bag error rate output from the *randomForest* package in R is an overall measure in optimizing the model, which is defined as the sum of the number of “false positive” and the number of “false negative” divided by the number of all estimation.

## Results

We obtained 82 person-days of data out of the expected 91 person-days. Of the 82 person-days, valid GPS data covered about 81.4% of the intended observation time. The main reason for the missing GPS points was the limited battery run time. We did not consider the missing data when developing the model. The missing data were expected to be proportionally distributed for each time-activity category and have little impact on the model performance; however, it was hard to obtain the exact distribution of the missing data. Overall we obtained 338,497 GPS points at the 15 seconds interval after removing erroneous points. On average, the study participants spent 88.3%, 1.1%, 2.5%, and 6.9% of time under the indoor, outdoor static, outdoor walking, and in-vehicle travel conditions, respectively. The study subjects spent 1.2% of time for other activities (e.g. traveling by bicycle, bus), which were excluded from model development.

### Model parameter tuning

The out-of-bag error decreased rapidly with the increase in the number of trees, but became stable when the tree number reached about 40. Considering the negligible gain in further increasing the number of trees, we selected 100 trees in the model which was assumed to a good tradeoff between the model precision and the computational time.

The out-of-bag errors dropped when the number of candidate predictors increased for outdoor static, outdoor walking, and in-vehicle travel conditions, but rose slightly for indoor conditions. Five to seven candidate predictors on each node of a decision tree seemed to reach a good balance of precision for all time-activity categories.

### Variables selection

**Fig 3:**
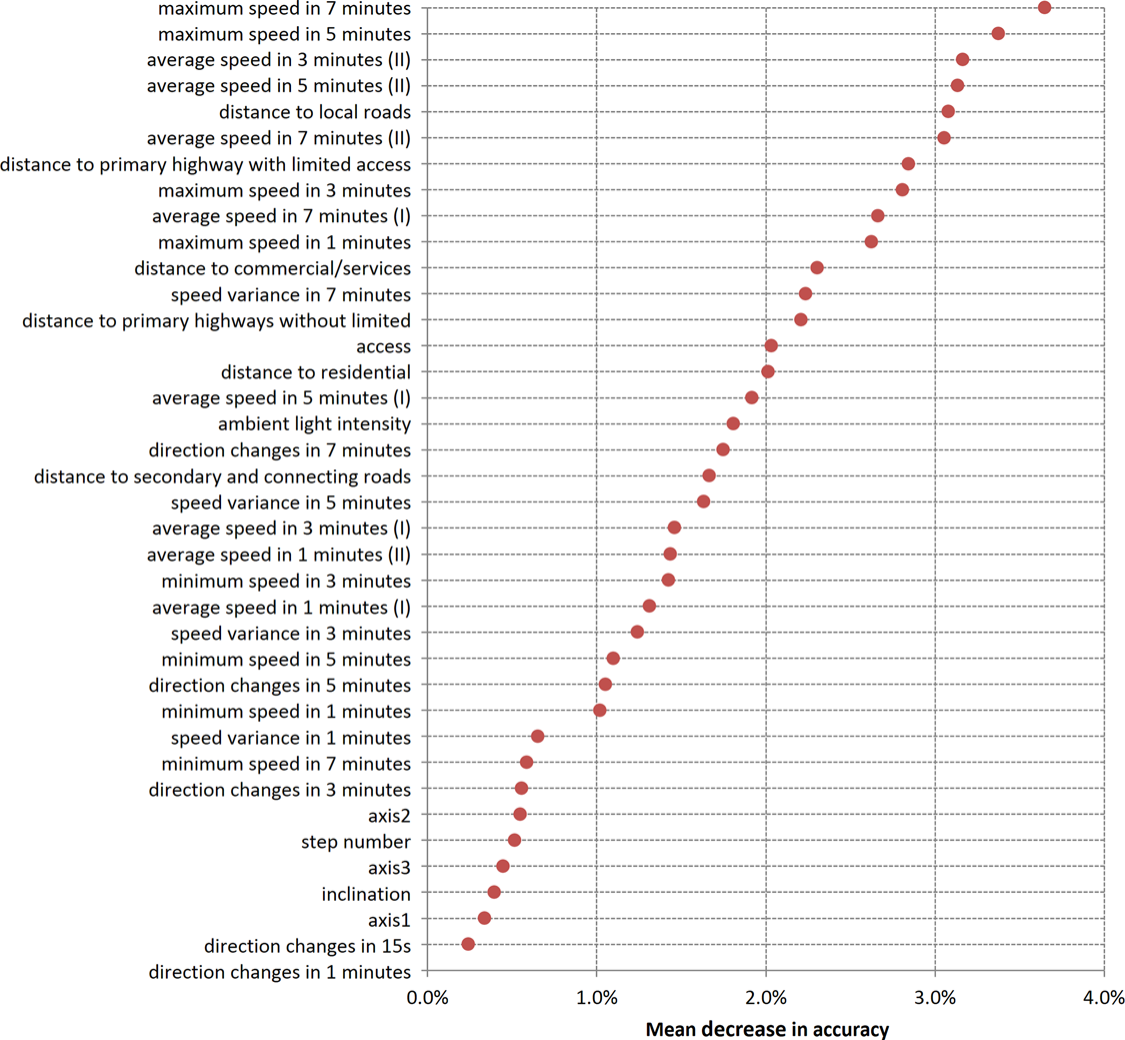
Mean decrease in accuracy for candidate variables.

Fig 3 shows the descending order of variables by their MDA values from top to bottom. A high MDA value indicates high importance of the variable or a large accuracy loss when the variable is excluded from the model. The most important variable was found to be maximum speeds [in 7 and 5 minutes], followed by average speeds [in 3 minutes (type-II speed), 5 minutes (type-II speed), distance to the local roads, average speeds in 7 minutes (type-II speed), distance to primary highway with limited access, maximum speed in 3 minutes, and so on.

**Fig 4:**
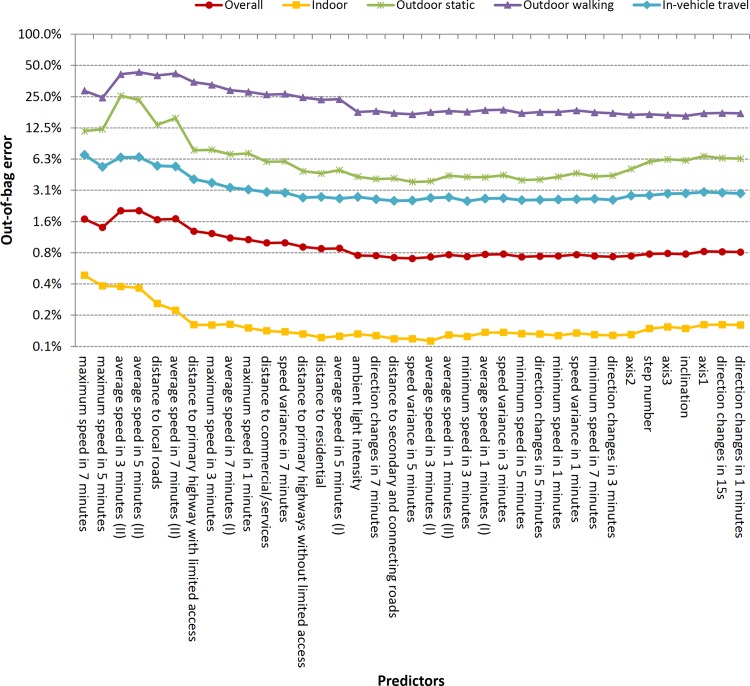
Out-of-bag error variation with different variables (from left to right the variables on the X axis were sequentially entered into the random forests model).

According to the importance ranking, variables were entered into the random forests models one by one from the most important to the least important. The overall and individual out-of-bag errors for each of the four time-activity category were calculated (Fig 4). The overall out-of-bag error decreased with added variables. Outdoor walking and indoor condition had the highest and lowest errors, respectively. Out of the 37 variables, we selected 15 variables that apparently decreased the overall out-of-bag error to build the random forests models: maximum speed in 1, 3, 5 and 7 minutes, average speed in 5 and 7 minutes based on accumulated distance during the average time, speed variance in 5 and 7 minutes, direction changes in 7 minutes, distance to primary highways, secondary and connecting roads, distance to residential and commercial/services, and ambient light intensity. Although the average speed (type II) in 3, 5 and 7 minutes, and distance to local roads decreased the out-of-bag error for indoor classification, they increased error for the other categories. So they were excluded from the final model. Among the six accelerometer variables, only ambient light intensity entered into the model. Besides distances to highways and local roads (FCC = A1-A4), we also analyzed distance to lower class roads (FCC = A5-A7). When the above ten variables were selected, distance to lower class roads improved the overall sensitivity and specificity by 0.2% and 0.1%, respectively. The sensitivity for outdoor static and outdoor walking improved by 1% and 2%, respectively, while the sensitivity and specificity for other categories changed to a much smaller extent. Therefore, we did not include distances to lower class roads in the final model.

### Model validation results

Table 1 shows the model validation results. With all the 15 variables and the leave-one-fold-out validation, we observed >99.00% accuracy and 96.33–99.98% specificity for all the four time-activity categories, and ≥97.55% sensitivity for all categories except outdoor walking (84.62%). The overall accuracy for all the categories was 99.71%. When ambient light intensity was excluded, sensitivity, specificity, and accuracy only dropped slightly for all the four time-activity categories except outdoor walking (sensitivity decreased from 84.62% to 80.94%). When both ambient light intensity and supplemental spatial variables (including distances to local roads and highways, and distances to residential and commercial/services parcels) were excluded, sensitivity dropped greatly for outdoor walking from 84.62% to 71.85%. Specificity for indoor time-activity also decreased moderately from 96.33% to 90.12%.

**Table 1:** Model validation of time-activity classification by leave-one-fold-out and leave-one-subject-out

Method	Predictor variables	Predicted vs. Actual	Indoor	Outdoor static	Outdoor walking	In-vehicle travel	Sensitivity	Specificity	Accuracy
Leave one fold out	With all 15 predictors	Indoor	283098	8	201	64	99.90%	96.33%	99.56%
		Outdoor static	94	5167	25	11	97.55%	99.98%	99.94%
		Outdoor walking	767	34	5643	225	84.62%	99.87%	99.55%
		In-vehicle travel	228	5	160	17288	97.78%	99.90%	99.78%
	Excluding lux variable	Indoor	283096	8	214	53	99.90%	95.79%	99.51%
		Outdoor static	92	5173	19	13	97.66%	99.99%	99.95%
		Outdoor walking	983	23	5398	265	80.94%	99.87%	99.47%
		In-vehicle travel	174	6	161	17340	98.07%	99.89%	99.79%
	Excluding more variables ^a^	Indoor	282902	23	312	134	99.83%	90.12%	98.91%
		Outdoor static	904	4350	23	20	82.12%	99.99%	99.69%
		Outdoor walking	1528	11	4792	338	71.85%	99.83%	99.23%
		In-vehicle travel	496	4	201	16980	96.04%	99.83%	99.62%
Leave one subject out	With all 15 predictors	Indoor	281745	968	1666	1158	98.67%	81.15%	96.90%
		Outdoor static	2359	1133	411	30	28.81%	99.64%	98.76%
		Outdoor walking	2534	162	3772	500	54.13%	99.23%	98.24%
		In-vehicle travel	1173	2	331	19766	92.92%	99.43%	98.99%
	Excluding lux variable	Indoor	277003	1302	1471	5761	97.01%	76.46%	94.93%
		Outdoor static	2564	1223	106	40	31.10%	99.58%	98.73%
		Outdoor walking	3844	21	2554	549	36.65%	99.38%	98.01%
		In-vehicle travel	1166	3	344	19759	92.89%	97.86%	97.53%
	Excluding more variables ^a^	Indoor	281267	2407	1146	717	98.50%	73.32%	95.95%
		Outdoor static	3546	239	59	89	6.08%	99.21%	98.06%
		Outdoor walking	3769	29	2560	610	36.74%	99.50%	98.12%
		In-vehicle travel	1270	33	357	19612	92.20%	99.52%	99.03%

^a^ excluding lux and supplemental spatial variables, i.e. distance to roadways and residential/commercial parcels.

Leave-one-subject-out validation showed worse model performance than the leave-one-fold out results. With all the 15 variables, we obtained 96.90%-98.99% accuracy, 28.81%-98.67% sensitivity, and 81.15%-99.64% specificity for the four time-activity categories using the leave-one-subject-out validation. The lowest sensitivity of 28.81% was observed for the outdoor static condition. The indoor condition had the highest sensitivity of 98.67%, but the lowest specificity of 81.15%. The overall accuracy for the four time-activity categories was 98.22%. When ambient light intensity was excluded, there was about 5% decrease in specificity for indoor condition. The sensitivity decreased as much as 17.48% for outdoor walking, but this was less severe for outdoor static condition. When both ambient light intensity and supplemental spatial variables were excluded, sensitivity for outdoor static condition dropped greatly from 28.81% to 6.08%. Sensitivity for outdoor walking and specificity for indoor condition also decreased largely.

## Discussion

We developed an automated time-activity classification method based on raw GPS data from participants under free living conditions and publicly-available roadway and land-use data. Our classification method used the random forests model with no user-defined rules, which makes it potentially adaptive to different study regions or populations. GPS path direction and distance to land-use data were included in the model, which to our knowledge has rarely been considered in previous studies.

GPS has been increasingly used in air pollution epidemiological research to track subjects’ time activities in different microenvironments where pollutant levels might vary greatly. User-defined rules based on physical principles and summary statistics have been used to classify major microenvironments [42–44, 61, 62]. These rules are usually interpretable, easily understood, and flexible in incorporating new knowledge, but a large challenge to the rule-based approach is the difficulty in determining threshold values for each rule. The threshold values may be influenced by local environment (e.g. building structure, density of high buildings, and land surface). For example, Oreskovic et al. (2012) classified the GPS points within 25 m of the center of the residence boundary as home [61], while Breen et al. (2014) defined home as the area within a 5 m buffer of the residence boundary [44]. A single threshold value may be efficient conceptually for a homogeneous environment in terms of buildings, facilities, and other spatial features, but is often not the case under the complex real-world situations. But the limited number of thresholds in few rules are usually not enough to represent the real-world conditions. The random forests model defines a large number of rules and threshold values in hundreds of trees. Thus the random forests model is not constrained by a few user-defined threshold values because of the large number of trees and the two levels of random selection process, which include random subset of input data for building trees and random selection of variables at split nodes. The model can be dynamically built and adapted for various research areas and populations.

Maximum speed, average speed, and distance to highways were the most important variables in the classification model. Among all the candidate variables, some of them were moderate or highly correlated (such as average speed in different time intervals). Thus the index of the mean decrease in accuracy might not reflect the real importance for some variables. However, the selected variables with high importance output from the model should contain the most necessary information to improve the model performance. Maximum and average speeds with different averaging times are helpful in distinguishing in-vehicle travel versus outdoor static or outdoor walking under different situations. For instance, speed averaged over a longer time (e.g. 7 minutes) is helpful in classifying GPS points near street intersections with stop sign or traffic light since usually a long-term average speed for in-vehicle travel would be higher than that of outdoor static and outdoor walking conditions. Whereas, the speed averaged over a shorter time (e.g. 3 minutes) is helpful in separating outdoor static or walking from in-vehicle travel for GPS points in a parking lot. Distance to highways can separate a large portion of indoor GPS points from in-vehicle travel points for houses far away from the roads. Direction change can capture scattered GPS points in or around a building [44]. Average direction changes can be viewed as a proxy measure of GPS accuracy due to multipath reflection and the quality of satellite signals. A large value in the average direction change likely indicates an indoor environment where GPS points drift frequently due to relatively poor satellite signal, while the direction change is usually small for in vehicle or walking activities. The scattered GPS points from low quality of satellite signals can also be captured by a spatial buffer. Nethery et al. (2014) assigned GPS points within 25 m and 5 m of subject’s home as “*Home*” for flat zone where the temperature changed by more than 0.1°C/minute and transition zone where the temperature was constant or changed slowly, respectively [43]. Besides the spatial buffer, Breen et al. (2014) used a 15 seconds temporal buffer to determine whether a GPS point within 15 seconds fell inside a spatially-buffered building boundary.

We used parcel-level land-use data instead of user-provided addresses or locations of residences, schools and working places [43, 44, 61]. With the user-provided addresses and the use of Google Earth, Breen et al. (2014) manually drew the rooftops for nine participants’ homes, workplaces and schools [44]. They obtained a high overall accuracy of 99.5%, a sensitivity from 60.4% to 100.0%, and >99.0% specificity when predicting eight categories of time-activity (e.g. indoors and outdoors at home, work, school; in-vehicle; other). Our model almost achieved comparable accuracy, sensitivity, and specificity without detailed individual-level address and building rooftop information. In addition, the number of our GPS points were about two times more than the number of their points, while we classified the points into four major time-activity categories. Without accelerometer variables, the performance of our model using GPS, roadway, and parcel land-use data did not decline greatly except for the decreased sensitivity for walking outside. When both addresses (e.g. home, school, work) and parcel data are available in the time-activity classification, we will likely be able to obtain more detailed time-activity categories and at a higher precision. Roadway data are usually publically available in urban areas. Currently, parcel-level data may not be widely available. But with the development in remote sensing and image processing technologies, high resolution land-use data will become more available globally [63].

Accelerometers have been widely used to study physical activity [62, 64, 65]. However, the importance of accelerometer measures (e.g. axis 1–3, step number, and inclination) by the mean decrease in accuracy was lower than that of the other GPS and spatial variables in the research (Fig 3). This was because some of the information provided by the accelerometer data may already be reflected in the GPS and/or other spatial variables. For instance, walking can be reflected by GPS speed. Average direction may reflect indoor environment where accelerometer-based physical activity level is low. Ambient light intensity had a moderate effect among all included variables in the model. We compared the model results with and without light sensor variables. With the other GPS and spatial variables in the model, light sensor variables only slightly improved the model performance (about 4.0% increase for the sensitivity of outdoor walking, whereas about 0.0% to 0.5% of improvement for other time-activity categories and other model performance measures). On the other hand, light sensor data are associated with uncertainties and errors because the device can be easily affected by outdoor environments (e.g. weather) and subjects may not wear it properly or follow protocols strictly [40, 41]. Therefore, the use of accelerometer might not be cost-effective for tracking only time-activity patterns for a large number of participants when high quality GPS and other spatial variables are available.

Without the light sensor variable, the other fourteen variables in the model can be easily computed from raw GPS points, roadway, and land-use data. GPS tracking and recording capabilities are currently available in most smartphones. By the end of 2014, about 25% of the world’s total population use smartphones, and the percentage may reach about 34% by 2017 [66]. It is estimated that 15 countries worldwide will have more than 50% their population use smartphones [66]. Smartphone might be the best choice for tracking time-activity patterns of a large population. At the same time, the latest smartphones by themselves have many sensors, such as accelerometer and light sensors, which are useful to record more environmental and behavioral information. Currently, battery life may be one of the biggest problems of smartphone use [45, 67]. Wu et al. (2010) reported less than 9 hours of battery life of a smartphone when it was turned on to record GPS data [45]. We expect the battery life of smartphones will improve in the near future.

Since the number of four categories were unbalanced in the data, the reliability of measures for each category might be different [68]. However, in this study we did not consider the influence of the respective sizes of individual time-activity categories. The leave-one-subject-out model validation showed different results compared to the commonly-used leave-one-fold-out model validation (Table 1). The largest difference between the two validation methods was observed for the sensitivity for outdoor static and outside walking conditions. The possible reason might be that the activity patterns of some subjects were significantly different from those of the other subjects. When a subject was left out, the model trained by the other subjects could not capture the particular activity patterns of this subject. The problem can be minimized by including a diverse population of subjects who conduct as many types of activities as possible. Caution is warranted when using the model trained from a limited number of subjects or unrepresentative sample to a large population.

There are three limitations in this study. First, the participants were convenient samples of students and staff from University of California, Irvine, and the sample size was small. Although the GPS points almost covered the whole study area, the student participants may have relatively simple life style on campus. A more general population with diverse socio-demographic characteristics can be involved in the future to collect more comprehensive time-activity data. Since several other activity modes, such as bicycle and bus, were not included in the study, our data might just represent a limited number of people with similar environment and life style. More subjects and activity modes need to be considered in future research. Furthermore, we believe that the method of machine learning is generalizable to different types of data, including the data with bicycle, bus, and light rail travels. The second limitation is the missing GPS data due to some incidents during data collection (e.g. the device ran out of battery). The missing data might not be proportionally distributed among the four considered categories. The third limitation is that the study area was mainly in Southern California. Results from this region might not be generalizable to other regions. Nevertheless, researches from other regions can adapt our modeling approach and develop their own models using locally-collected GPS time-activity tracking data.

## Conclusion

We successfully developed an adaptive time-activity classification model based on a decision tree approach and GPS tracking data from free living subjects. The model was able to classify four major time-activity categories (i.e. indoor, outdoor static, outdoor walking and in-vehicle) to a satisfactory accuracy level. The sensitivity of the model in identifying outdoor walking was lower than the other categories. High classification accuracy can be obtained by using just raw GPS data and publicly-available spatial data (i.e. roadway and land-use data). However, we should be cautious when generalizing the model developed from a small number of subjects to other population. The methodology we developed can be utilized in other studies to classify time-activity categories based on GPS data.
